# The effects of continuing aspirin and clopidogrel on perioperative outcomes in primary elective total knee and hip replacement: A systematic review and meta-analysis

**DOI:** 10.1016/j.jor.2025.07.024

**Published:** 2025-07-24

**Authors:** M.M. Farhan-Alanie, A. Abdul-Hussein, A. Stephens, M. Blankstein

**Affiliations:** a Warwick Medical School, University of Warwick, Coventry, CV2 2DX, United Kingdom; b Faculty of Medicine, Imperial College London, London, SW7 2AZ, United Kingdom; c Department of Trauma & Orthopaedics, University Hospitals Coventry & Warwickshire NHS Trust, CV2 2DX, United Kingdom; d Department of Orthopaedics and Rehabilitation, University of Vermont Larner College of Medicine, Burlington, VT, United States of America

**Keywords:** Antiplatelet, Aspirin, Clopidogrel, THR, TKR

## Abstract

**Introduction:**

Approximately 20 % of patients undergoing primary elective total hip (THR) or knee replacement (TKR) take an antiplatelet medication. The two main management strategies are continuing or discontinuing the antiplatelet medication pre-operatively. Continuing antiplatelets may increase the risk of bleeding, wound complications, and infection. Discontinuing antiplatelets may increase the risk of cardiac and cerebrovascular adverse events. This systematic review and meta-analysis evaluated the effects of continuing aspirin or clopidogrel on the perioperative outcomes of patients undergoing THR or TKR.

**Methods:**

Medline, Embase, Web of Science, and Cochrane Library were searched for randomised controlled trials and cohort studies reporting on outcomes blood loss, infection, wound complications, operative duration, length of stay, venous thromboembolism, cardiac and cerebrovascular events, mortality, and others. Random effects meta-analysis was performed.

**Results:**

Eight studies enabled inclusion of data on 928 THRs and 3526 TKRs. Continuing antiplatelet therapy did not affect intraoperative blood loss in THR (MD -16.57 ml, 95 % CI -120.75–87.61, p = 0.76) or TKR (MD -0.06 ml, 95 %CI -6.04–5.91, p = 0.98). However, TKR patients continuing antiplatelet therapy had a higher risk of blood transfusion (OR 1.63, 95 %CI 1.25–2.13, p = 0.0003) although there were no differences observed in THR patients (OR 1.71, 95 %CI 0.84–3.49, p = 0.14). No significant differences were found for outcomes infection, cardiac and cerebrovascular events, and post-operative mortality between patient groups following THR and TKR.

**Conclusions:**

Continuing antiplatelet use during TKR, but not THR, was associated with an increased risk of blood transfusion. Surgical complications and medical adverse events were not influenced by antiplatelet use. Further research with larger sample sizes is needed to draw definitive conclusions.

PROSPERO (CRD42024470601)

## 1 Introduction

Increased life expectancy, multimorbidity, and polypharmacy have contributed to the high prevalence of patients taking an antiplatelet medication.^1^, ^2^, ^3^ In the United States in 2019, over one-third of adults aged 40 years and older used aspirin for primary or secondary cardiovascular disease prevention, and this figure is predicted to rise.^4^ Among those undergoing elective primary total knee replacement (TKR) and total hip replacement (THR), approximately 15 %–25 % are on antiplatelet therapy.^5^^,^^6^ This reflects a large absolute number of patients and understanding the optimal perioperative management of antiplatelet medications in these patients is crucial. Furthermore, due to an aging population and the consequent increase in the prevalence of hip and knee osteoarthritis, a growing proportion of these patients will be taking antiplatelet medications.^7^ The two primary management strategies are either continuing or discontinuing the antiplatelet medication during the perioperative period, each associated with different risk profiles. Continuing antiplatelet therapy may increase the risk of bleeding, the need for allogenic blood transfusion, wound complications, and surgical site infection.^8^ Conversely, discontinuing antiplatelets pre-operatively may increase the risk of cardiac and cerebrovascular adverse events, as well as mortality.^9^ These risks are exacerbated by a rebound prothrombotic state that occurs in the initial period following antiplatelet discontinuation.^10^

Guidelines from the American Academy of Orthopaedic Surgeons (AAOS) recommend that patients discontinue antiplatelet medications, such as aspirin and clopidogrel, before elective hip or knee arthroplasty.^11^ However, this recommendation was based on studies that were not specific to elective hip or knee arthroplasty procedures. Since publication of this guideline in 2011, several studies addressing this topic involving patients undergoing arthroplasty have been published. This systematic review and meta-analysis aims to evaluate the effects of continuing aspirin or clopidogrel on the perioperative outcomes of patients undergoing elective primary unilateral THR and TKR, in comparison to those who discontinued these medications pre-operatively or were not previously prescribed them.

## 2 Methods

### 2.1 Data sources and search strategy

The study adhered to PRISMA (Preferred Reporting Items for Systematic Review and Meta-Analysis) and MOOSE (Meta-Analysis of Observational Studies in Epidemiology) guidelines.^12^^,^^13^ Medline, Embase, Web of Science, and Cochrane Library were searched from their inception to August 16, 2024. Table S1 details the search strategy. Search results were independently assessed for inclusion by two authors (MFA, AAH). Initial screening was by title and abstract. Further screening of selected full texts determined eligibility. Bibliographies of included articles were manually scanned to identify missed relevant articles. The study was registered in PROSPERO (CRD42024470601).

### 2.2 Eligibility criteria

Randomised controlled trials (RCTs) and comparative observational studies were eligible for inclusion. Eligible studies included those examining patients undergoing primary elective unilateral THR or TKR and compared those who continued taking aspirin or clopidogrel (antiplatelet group) to those who discontinued these medications or were not taking them before their surgery (non-antiplatelet group). The outcomes of interest are listed in Table 1. Where studies included the results of both patients who discontinued the medications and those who were not previously taking them, data of the former group was preferentially selected for inclusion in the analysis. Data from studies that included revision or bilateral procedures were excluded. Studies examining the effectiveness of initiating antiplatelet medications pre-operatively for prophylaxis against specific conditions such as venous thromboembolism were excluded. Studies in English or with an accessible translation were included. Disagreements regarding study inclusions and marginal studies for eligibility were discussed with the senior author (BLINDED).

**Table 1:** Outcomes under investigation.

Surgical Outcomes
Volume of blood loss (calculated, intraoperative, post-operative surgical drain)
Blood transfusion
Length of procedure
Tourniquet time
Surgical site infection
Wound complications
Length of hospital stay
Re-admission
Re-operation
*Medical Outcomes*
Venous thromboembolism
Cardiac events
Cerebrovascular events
Mortality

### 2.3 Data extraction and quality assessment

Data was independently extracted using a standardized form, and the methodological quality of the studies were assessed using the Newcastle-Ottawa Scale by two authors (BLINDED, BLINDED).^14^ This assesses three domains (selection, comparability, and outcome) across eight items with each scoring one point except for comparability which can be adapted to the specific topic to score up to two points. The two risk factors selected *a priori* to be most pragmatic and relevant to control for confounding between the comparison groups were body mass index and American Society of Anaesthesiologists grade. Studies scoring less than five points were judged as high risk of bias and excluded, five to seven were considered medium risk, and eight or nine points were deemed low risk.

### 2.4 Data synthesis and analysis

The meta-analysis was conducted using Review Manager 5.3 (RevMan Version 5.3. Copenhagen: The Nordic Cochrane Centre, The Cochrane Collaboration, 2014). An inverse variance method was used and random-effects models were applied. Summary measures were abstracted from papers as mean differences (MD) for continuous outcomes and odds ratios (OR) for binary outcomes, along with 95 % confidence intervals (CI). Risk differences (RD) were used to report outcomes when no events occurred in either arm of one or more studies within a comparison. Higgins I^2^ test was performed to provide an estimate of the extent of statistical heterogeneity. 95 % confidence intervals (CI) were calculated for each study with statistical significance set at p ≤ 0.05. Summary estimates of the overall effect are provided as a forest plot. Narrative discussion has been provided where statistical analysis was not possible.

## 3 Results

### 3.1 Study identification and selection

**Fig. 1:**
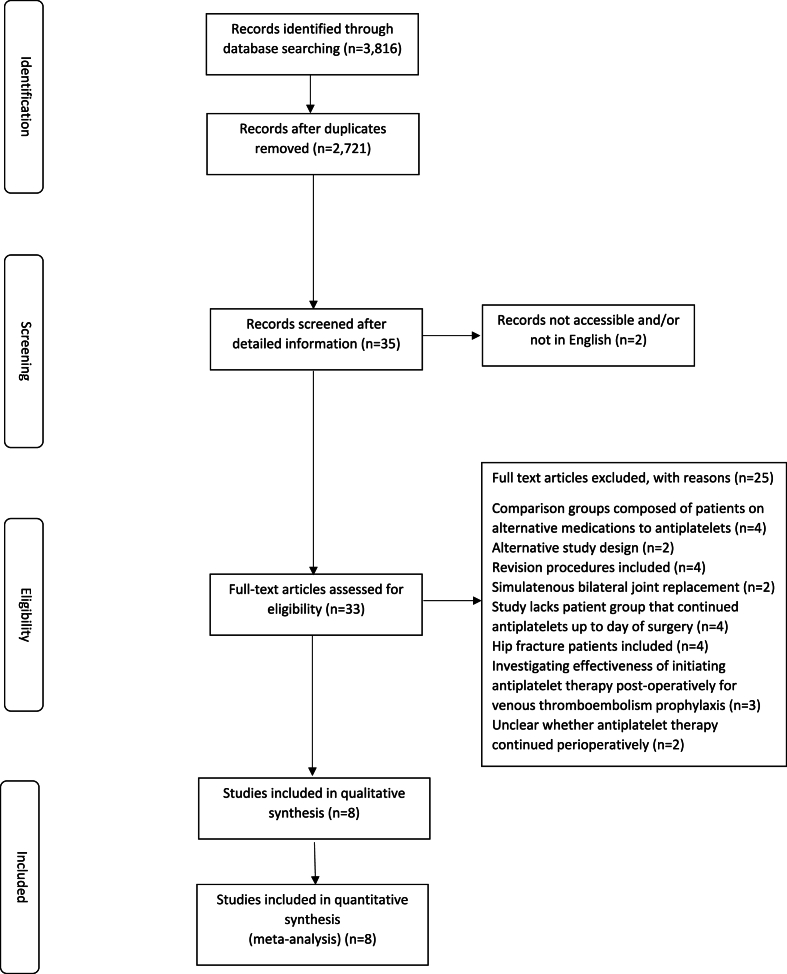
PRISMA flow diagram.

The literature search identified 2721 articles. Fig. 1 illustrates the study selection process. Eight publications met the inclusion criteria (Table 2).^6^^,^^15^, ^16^, ^17^, ^18^, ^19^, ^20^, ^21^

**Table 2:** Description of included articles.

Publication Year	First Author	Study Design	Study Time Period	Country	Age (Years)	Sex (Male:Female) (%)	Procedures	Antiplatelet	Timing of pre-operative discontinuation	Total Procedures (continued/discontinued or control)	Outcome(s) Investigated
**2012**	Cossetto	Cohort Study	2008–2010	Australia	Range 62–91	43.2:56.8	50 THR and 89 TKR	100 mg Aspirin OD	N/A	63/76	**Intraoperative blood loss, post-operative blood loss** (suction drains), **operative time, pulmonary embolism,**
**2019**	Chen	Cohort Study	December 2010–December 2012	Taiwan	Range of mean values 71.6 to 74.2	22:78	1295 TKR	100 mg (84.8 %) or 325 mg (15.2 %) Aspirin OD	N/A	217/1078	**Perioperative blood loss** (calculated blood loss, transfusion rate, transfusion volume), **tourniquet time, length of hospital stay, deep vein thrombosis, pulmonary embolism, cardiac and cerebral complications**
**2020**	Ashkenazi	Cohort study	2011–2018	USA^a^	range of mean values 60.6 to 71.9	38.7:61.3	757 THR	100 mg Aspirin OD	N/A	205/552	**Perioperative blood loss** (transfusion rate), **30-day and one-year readmission, haematoma formation, wound discharge, superficial and deep surgical site infection, mortality**
**2016**	Meier	Cohort study	October 2007–September 2009	Switzerland	Range of mean values of THR groups 74.3 to 78.3	51.9: 48.1 (THR)	79 THR and 96 TKR	100 mg Aspirin (most common dose)	10 days before surgery	19/60 (THR)	**Intraoperative blood loss** (suction and swab volume), **post-operative blood loss** (suction drains), **perioperative blood loss** (volume of blood transfusion), **haematoma following THR, infection, cardiac and cerebral complications** (up to four weeks post-operatively)**, knee range of motion, PROMs** (WOMAC at hospital discharge, eight weeks, and one year post-operatively), **mortality**
					Range of mean values of TKR groups 70.9 to 72.2	53.1: 46.9 (TKR)				17/79 (TKR)	
**2022**	Li	Cohort study	January 2014–December 2019	China	Range of mean values 69.62 (6.43) and 70.39 (5.94)	24.8:75.2	560 TKR	100 mg Aspirin (80.71 %)	N/A	180/380	**Intraoperative blood loss, post-operative blood loss** (suction drains), **perioperative blood loss** (transfusion rate, transfusion volume), **length of hospital stay, tourniquet time, cardiac and cerebral complications**
								75 mg Aspirin (19.29 %)			
**2017**	Schwab	Cohort study	2006 to 2014	Belgium	Overall mean 67 (SD 10)	35.1: 64.9	455 TKR	Majority taking 80 mg Aspirin	N/A	154/301	**Perioperative blood loss** (transfusion rate, transfusion volume), **length of hospital stay, operative time, tourniquet time**
**2020**	Hang	Cohort study	March 2011 to March 2016	Singapore	Median 68 years in both groups	23.56:76.44	777 TKR	100 mg Aspirin	N/A	77/700	**Perioperative blood loss** (calculated blood loss, transfusion rate), **length of hospital stay**
**2022**	Wu	Cohort study	January 2009 to December 2018	Taiwan	Range of mean values 71.5 (range 40–85) to mean 72.6 (range 50–90)	46.6:53.4	42 THR and 254 TKR	Clopidogrel	>5 days before surgery	15/27 (THR)	**Perioperative blood loss** (estimated blood loss, transfusion rate, transfusion volume), **deep surgical site infection, cardiac and cerebral complications, deep vein thrombosis, pulmonary embolism, 30-day readmission, reoperation, mortality**
										41/213 (TKR)	

# Not reported.

a United States of America.

### 3.2 Study characteristics and study quality

All included studies were observational by design. There were data on 928 THRs and 3526 TKRs. Two studies assessed the outcomes of patients who continued the use of antiplatelets compared to those who discontinued them pre-operatively^19^^,^^21^ while six studies involved comparison to a group that were not previously taking antiplatelets.^6^^,^^15^^,^^16^^,^^18^^,^^20^^,^^22^

Table S2 details the results of the bias assessment.

### 3.3 Blood loss

#### 3.3.1 Intraoperative blood loss

**Fig. 2:**
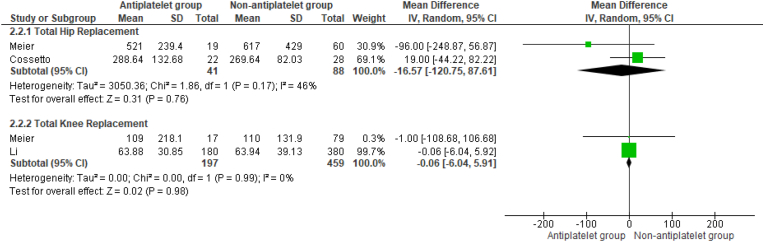
Mean differences in intraoperative blood loss between patient groups who underwent THR and TKR.

Pooled analysis found no differences in intraoperative blood loss between groups during both THR and TKR (MD -16.57 mL, 95 % CI -120.75 to 87.61, p = 0.76, and MD -0.06 mL, 95 %CI -6.04 to 5.91, p = 0.98, respectively) (Fig. 2).

#### 3.3.2 Post-operative blood loss

**Fig. 3:**
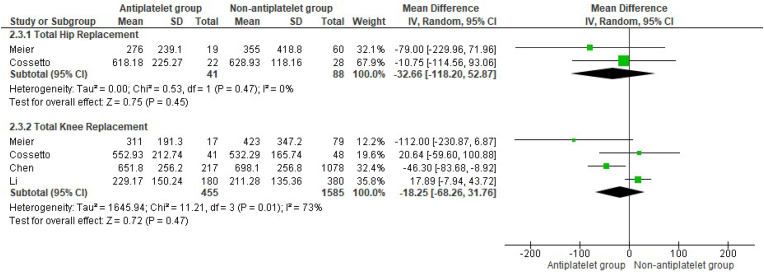
Mean differences in post-operative blood drainage volumes between patient groups who underwent THR and TKR.

Post-operative surgical drain volumes following THR and TKR were not significantly different between groups (MD -32.6 mL, 95 %CI -118.20 to 52.87, p = 0.45, and MD -18.25 mL, 95 %CI -68.26 to 31.76, p = 0.47 respectively) (Fig. 3).

### 3.4 Allogenic blood transfusion

**Fig. 4:**
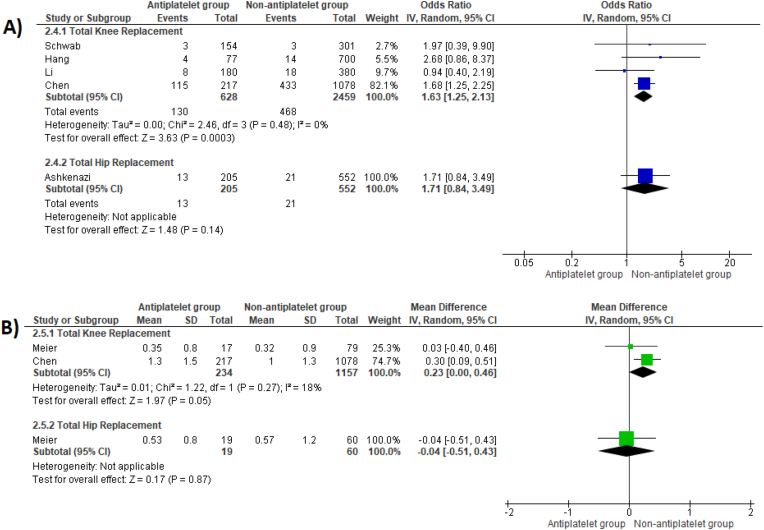
Odds ratios comparing risk of receiving peri-operative blood transfusion (A) and mean differences in number of units of blood transfused (B) between patient groups for both THR and TKR surgery.

Pooled analyses found no significant differences in receipt of a blood transfusion (OR 1.71, 95 %CI 0.84 to 3.49, p = 0.14) or transfused volume (MD -0.04 units, 95 %CI -0.51 to 0.43, p = 0.87) between patient groups following THR (Fig. 4). However, TKR patients who continued antiplatelet therapy experienced a significantly higher transfusion risk (OR 1.63, 95 %CI 1.25 to 2.13, p = 0.0003) and were transfused a greater volume (MD 0.23 units, 95 % CI 0.00 to 0.46, p = 0.05) (Fig. 4).

Wu et al. conducted a combined analysis of THR and TKR operations. They found no significant differences between the two groups for allogenic blood transfusion (19.6 % versus 16.7 %, p = 0.28) or volume transfused (2.4 ± 0.8 versus 2.4 ± 1.1 units; p = 0.85).

Meier et al. reported that perioperative transfusion did not significantly differ between groups for both THR and TKR patients.

### 3.5 Calculated blood loss

Pooled analysis found no significant differences in calculated blood loss between groups for TKR (MD 39.49 mL, 95 %CI -28.87 to 107.84, p = 0.26) (Fig. S1). Similarly, Hang et al. found no differences between patient groups; median 480 ml (interquartile range 298–636) versus 468 ml (interquartile range 318–603), p = 0.724. However, Wu et al. found estimated blood loss was relatively greater among patients taking clopidogrel and undergoing either TKR or THR surgery (mean 1212.3 mL versus 1068.9 mL, p < 0.03).

### 3.6 Infection

**Fig. 5:**
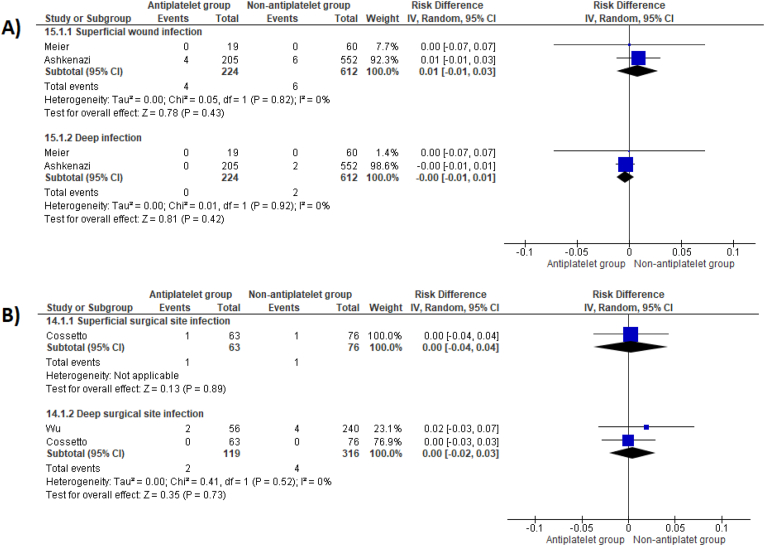
Risk differences for superficial and deep surgical site infection between patient groups following THR (A) and following either THR or TKR surgery (B).

There were no differences in superficial and deep surgical site infection between patient groups who underwent THR surgery at one-year post-operatively (RD 0.01, 95 %CI -0.01 to 0.03, p = 0.43, and RD 0.00, 95 %CI -0.01 to 0.01, p = 0.42 respectively) (Fig. 5A). Meier et al. reported no surgical site infections occurred in either TKR patient group at one year post-operatively (0/17 versus 0/79).

Pooled analysis of studies reporting on both THR and TKR procedures found no difference in superficial and deep surgical site infection (RD 0.00, 95 %CI -0.04 to 0.04, p = 0.89, and RD 0.00, 95 %CI -0.02 to 0.03, p = 0.73, respectively) (Fig. 5B).

### 3.7 Wound complications

Ashkenazi et al. reported no significant differences in the proportion of patients who experienced post-operative haematomas (2 % versus 2.7 %, p = 0.79) and persistent wound discharge within and after four days following THR (5.4 % versus 6.3 %, p = 0.64, and 7.1 % versus 11.7 %, p = 0.06, respectively). Chen et al. investigated minor wound complications following TKR (including excessive bullae formation, delayed wound healing, and superficial wound infection). They found no differences between groups at one year post-operatively (1.8 % versus 1.6 %, p = 0.478). Cosetto et al. reported none of the patients who underwent THR and TKR experienced wound dehiscence. Similarly, there were no differences in non-infective wound related complications at one year follow-up among TKR patients in the study by Li et al. (2.22 % versus 0.79 %, p = 0.309). Meier et al. found no differences in post-operative haematoma formation following THR (45.8 % versus 36.8 %, p = 0.495) and wound discharge seven days post-operatively following THR and TKR (45.8 % versus 31.6 %, p = 0.276, and 21.5 % versus 17.6 %, p = 0.721, respectively). Wound complications including prolonged discharge, blister formation, haemarthrosis, and skin necrosis were not significantly different between patient groups following TKR and THR in the study by Wu et al. (OR 1.55, 95 %CI 0.74 to 3.21, p = 0.23).

### 3.8 Length of procedure

**Fig. 6:**
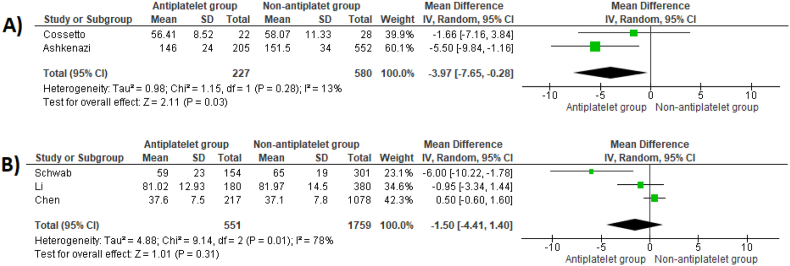
Mean difference in operative time between patient groups undergoing THR (A) and mean difference in tourniquet time between patient groups undergoing TKR (B).

THR patients who continued antiplatelet medications experienced relatively shorter operative times (MD -3.97 min, 95 %CI -7.65 to −0.28, p = 0.03) (Fig. 6A). For TKR, only Schwab et al. reported on length of procedure and found no difference between patient groups (MD -3 min, 95 %CI -6.90 to 0.90, p = 0.13). However, several articles on TKR tourniquet time. Pooled analysis demonstrated no differences between groups (MD -1.50 min, 95 %CI -4.41 to 1.40, p = 0.31) (Fig. 6B).

### 3.9 Length of hospital stay

**Fig. 7:**
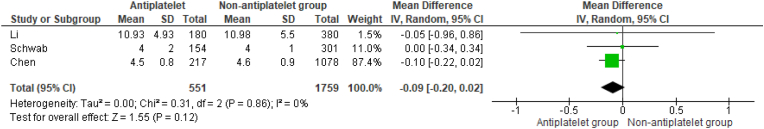
Mean difference in length of hospital stay between patient groups who underwent TKR surgery.

Pooled analysis found no differences in length of hospital stay between patient groups for TKR (MD -0.09 days, 95 %CI -0.20 to 0.02, p = 0.12) (Fig. 7). Similarly, Hang et al. found no differences in length of hospital stay between patient groups who underwent TKR (median 3 days, IQR 3 to 5 versus 3 days, IQR 3 to 4, p = 0.869). Wu et al. also reported no significant differences following THR and TKR (mean 4.5 days, range 2–10 versus mean 5.2 days, range 2–27, p = 0.16).

### 3.10 Venous thromboembolism

**Fig. 8:**
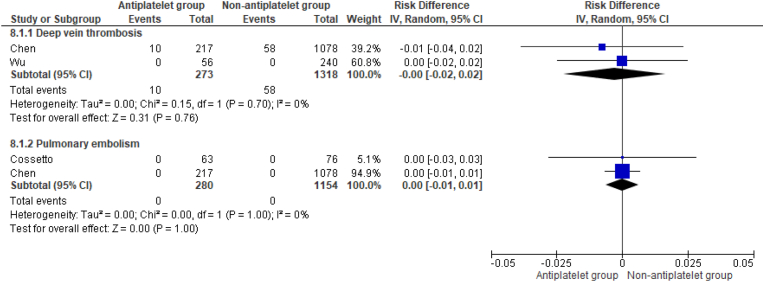
Risk differences in incidence of deep vein thrombosis and pulmonary embolism following TKR.

Pooled analysis demonstrated no differences in the incidence of deep vein thrombosis (DVT) and pulmonary embolism (PE) between patient groups undergoing TKR at minimum of 90 days post-operatively (RD 0.00, 95 %CI -0.02 to 0.02, p = 0.76, and RD 0.00, 95 %CI -0.01 to 0.01, p = 1.00, respectively) (Fig. 8). Li et al. evaluated venous thromboembolism as a composite outcome (DVT and PE) and found no differences at one year post-operatively following TKR (3/180 versus 17/380, p = 0.570).

Wu et al. reported that none of the THR patients in their study experienced a DVT at 90 days post-operatively (0/15 versus 0/27). However, one patient in the non-antiplatelet group experienced a PE (0/56 versus 1/240). It is not known whether this occurred in a patient who had undergone THR or TKR.

Cossetto et al. analysed outcomes of both THR and TKR procedures together and reported that three patients in each group developed a DVT (3/63 versus 3/76) however none experienced a PE.

### 3.11 Re-admissions and re-operations

Ashkenazi et al. reported on all-cause 30-day and operation-related one-year hospital re-admission following THR and found no difference between patient groups (15/552 versus 5/205, and 5/552 versus 3/205, p = 0.78, respectively). Similarly, Wu et al. found no differences in return to the emergency room within 30 days of THR or TKR surgery (OR 0.86, 95 %CI 0.36 to 2.07, p = 0.83). The authors also found no differences in return to the operating room (OR 4.35, 95 %CI 0.27 to 7.1, p = 0.34).

### 3.12 Cardiac and cerebral adverse events

Four studies reported on cardiac and cerebral adverse events.

Two of these studies made comparisons to patients who discontinued antiplatelet pre-operatively. Meier et al. reported no differences between patient groups for cardiac and cerebral complications following THR (0 % versus 11.7 %, p = 0.119, and 5.3 % versus 1.7 %, p = 0.392, respectively) and TKR (0 % versus 2.5 %, p = 0.507, and 0 % versus 0 %, respectively) at one-year post-operatively. Wu et al. analysed THR and TKR procedures together, and found no differences in transient ischaemic attacks and strokes (0/56 versus 6/240) and acute myocardial infarction (1/56 versus 7/240) between patient groups (OR 0.32, 95 %CI 0.04 to 2.48, p = 0.48).

Two studies compared results to patients who were not previously taking antiplatelet medications. Li et al. found no differences in cardiac (18.18 % versus 25 %, p = 0.944) and cerebrovascular events (9.09 % versus 12.50 %, p = 1.00) between TKR patient groups at one-year follow up. Chen et al. reported that none of the TKR patients experienced a cardiac or cerebrovascular event at one-year post-operatively (0/217 versus 0/1078).

### 3.13 Mortality

Wu et al. compared results to patients who discontinued their antiplatelet medication pre-operatively. They found no significant differences in 90-day post-operative mortality between patient groups undergoing either THR or TKR (0/56 versus 1/240). Chen et al. reported there was no in-hospital mortality between patient groups who underwent TKR (0/217 versus 0/1078). Ashkenazi et al. reported on mortality at one month and one-year following THR and found no differences between groups (1/205 versus 1/552, p = 0.47, and 4/205 versus 3/552, p = 0.40, respectively).

## 4 Discussion

This systematic review and meta-analysis assessed the impact of continuing antiplatelet therapy during THR and TKR surgery on various perioperative outcomes, compared to patients who discontinued these medications pre-operatively or were not previously prescribed them. Our study found that patients undergoing TKR who continued their antiplatelet medications were more likely to receive an allogenic blood transfusion and a larger transfusion volume. However, no significant differences were observed in intraoperative blood loss, post-operative surgical drain volumes, or calculated blood loss between the patient groups. In contrast, no significant differences in these outcomes were observed between patient groups undergoing THR surgery. Except for a marginally shorter operative duration favouring patients continuing antiplatelets while undergoing THR, no differences were found in the other investigated outcomes for both THR and TKR, including surgical site infection, wound complications, tourniquet time, length of hospital stay, venous thromboembolism, readmission and reoperations, cardiac and cerebrovascular events, and mortality.

In other patient populations, such as those with hip fractures, evidence suggests that performing surgery including arthroplasty while antiplatelet effects are still active is associated with greater haemoglobin decline however does not influence the risk of blood transfusion. There were also no differences in length of hospital stay, reoperation, cardiac and cerebrovascular events, venous thromboembolism, major bleeding, or other wound-related complications.^23^ It is important to mention this patient cohort are typically frailer and have more comorbidities than patients undergoing elective TKR and THR. Also, hip fracture surgery encompasses various operations, each associated with different bleeding and transfusion risks.^24^ For these reasons, the results of these studies can not be generalised to elective THR and TKR patients. Similarly, the current AAOS guidelines on this topic recommend discontinuing antiplatelet medications before elective THR or TKR surgery however this is based on three non-arthroplasty studies.^11^ These found that preoperative antiplatelet use was associated with increased perioperative blood loss. However, these studies may now be considered outdated, as they were conducted between 18 and 30 years ago and do not reflect the advancements in current clinical practice including anaesthetic and surgical techniques, updates to blood transfusion policies, and use of tranexamic acid. Furthermore, these studies also had sample sizes ranging from 100 to 200 patients only, and were underpowered to detect adverse cardiac and cerebrovascular events as well as mortality. In contrast, a meta-analysis involving 50,279 patients investigated the impact of non-compliance or pre-operative discontinuation of aspirin among patients at risk for coronary artery disease found a three-fold increase in the risk of major adverse cardiac events (OR 3.14, 95 %CI 1.75 to 5.61, p = 0.0001). Moreover, this risk was magnified in patients with intracoronary stents (OR 89.78, 95 %CI 29.90 to 269.60).^25^

The strengths of our study include its focused evaluation of only patients undergoing elective primary unilateral THR or TKR. We also investigated several outcomes and pooled results to improve the precision of the effect estimate. Due to distinct bleeding risks and medical considerations between THR and TKR surgery, we analysed outcomes for these procedures separately. The inclusion of studies completed in several different countries strengthened its external validity.

Our study has limitations, particularly the observational design of the included studies, which may introduce confounding. Also, surgeons’ reasons underlying the decision to suspend or continue antiplatelet treatment is a source of confounding by indication, possibly reflecting differences in risk profiles among patients. However, this was mitigated by some studies that reported surgeons had consistently advised patients to continue antiplatelet medications without risk stratification.^16^ Due to the rarity of venous thromboembolism, mortality, and cardiac and cerebrovascular events, our results are underpowered to allow definitive conclusions to be drawn for these outcomes. Ideally, a large database study would be needed to investigate these outcomes. Some studies compared patients who were not previously taking antiplatelet medications, which may slightly impact the ability to interpret the findings of adverse event outcomes, though it allowed meaningful comparisons for other outcomes such as blood loss.

The available evidence suggests that continuing low-dose antiplatelet medications during elective primary unilateral THR surgery does not affect the outcomes investigated compared to patients who withheld these medications or were not taking them prior to surgery. In contrast, TKR patients who continued their antiplatelets were more likely to receive an allogenic blood transfusion and were also transfused larger volumes. Surgeons should consider this finding during their decision-making process in this clinical scenario. Although several factors could have contributed to this finding, delineating the exact causes is not possible due to the inherent limitations of observational study designs. Further research with larger sample sizes is urgently needed to draw definitive conclusions particularly regarding adverse events. RCTs on this topic, though not specific to TKR and THR patients, have been performed, demonstrating they are feasible to conduct in this context to help answer this research question.^26^

## CRediT authorship contribution statement

**M.M. Farhan-Alanie:** Conceptualization, Methodology, Investigation, Data curation, risk of bias assessment, Formal analysis, Visualization, Writing – original draft, Writing – review & editing, Project administration. **A. Abdul-Hussein:** Data curation, risk of bias assessment. **A. Stephens:** Writing – review & editing. **M. Blankstein:** Supervision, Writing – review & editing.

## Patient consent

Not applicable as data from published sources.

## Ethical statement

Not required/applicable.

## Ethical statement

Not required/applicable.

## Funding statement

There were no sources of funding for this work.

## Declaration of competing interest

The authors report no conflicts of interest.
