# Supplementary material for: The effects of continuing aspirin and clopidogrel on perioperative outcomes in primary elective total knee and hip replacement: A systematic review and meta-analysis
Source: J Orthop. 2025 Jul 24;67:369–77. doi: 10.1016/j.jor.2025.07.024 (PMC12320541; doi:10.1016/j.jor.2025.07.024)
Supplement: Multimedia component 1 [file mmc1.docx]

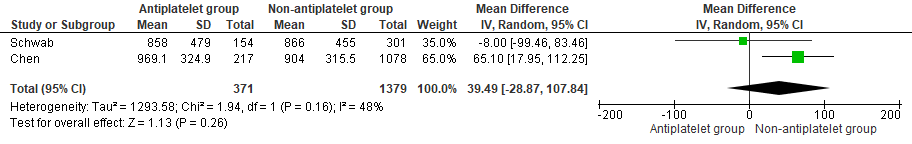
Figure S1: Mean differences in calculated blood loss between patient groups following TKR surgery
