# Supplementary material for: The effects of continuing aspirin and clopidogrel on perioperative outcomes in primary elective total knee and hip replacement: A systematic review and meta-analysis
Source: J Orthop. 2025 Jul 24;67:369–77. doi: 10.1016/j.jor.2025.07.024 (PMC12320541; doi:10.1016/j.jor.2025.07.024)
Supplement: Multimedia component 2 [file mmc2.docx]

| Medline and Embase | 1. antiplatelet.kw,ti,ab.  2. Platelet Aggregation Inhibitors/  3. Arthroplasty, Replacement, Hip/  4. Arthroplasty, Replacement, Knee/  5. ((Hip* or knee* or joint*) adj3 (replace* or arthroplast* or prosth* or endoprosth*)).ab. or ((Hip* or knee* or joint*) adj3 (replace* or arthroplast* or prosth* or endoprosth*)).ti.  6. aspirin/  7. clopidogrel/  8. ticagrelor/  9. prasugrel/  10. Cilostazol/  11. Dipyridamole/  12. 1 or 2 or 6 or 7 or 8 or 9 or 10 or 11  13. 3 or 4 or 5  14. aspirin.kw,ti,ab.  15. clopidogrel.kw,ti,ab.  16. ticagrelor.kw,ti,ab.  17. prasugrel.kw,ti,ab.  18. Cilostazol.kw,ti,ab.  19. Dipyridamole.kw,ti,ab.  20. 12 or 14 or 15 or 16 or 17 or 18 or 19  21. 13 and 20 |
| --- | --- |
| The Cochrane Library | #1 (antiplatelet):ti,ab,kw  #2 MeSH descriptor: [Platelet Aggregation Inhibitors] explode all trees  #3 MeSH descriptor: [Arthroplasty, Replacement, Knee] explode all trees  #4 MeSH descriptor: [Arthroplasty, Replacement, Hip] explode all trees  #5 (((Hip* or knee* or joint*) near/3 (replace* or arthroplast* or prosth* or endoprosth*))):ti,ab,kw  #6 (aspirin):ti,ab,kw  #7 (clopidogrel):ti,ab,kw  #8 (ticagrelor):ti,ab,kw  #9 (prasugrel):ti,ab,kw  #10 (cilostazol):ti,ab,kw  #11 (dipyridamole):ti,ab,kw  #12 MeSH descriptor: [Aspirin] explode all trees  #13 MeSH descriptor: [Clopidogrel] explode all trees  #14 MeSH descriptor: [Ticagrelor] explode all trees  #15 MeSH descriptor: [Prasugrel Hydrochloride] explode all trees  #16 MeSH descriptor: [Cilostazol] explode all trees  #17 MeSH descriptor: [Dipyridamole] explode all trees  #18 #1 or #2 or #12 or #13 or #14 or #15 or #16 or #17  #19 #3 or #4 or #5  #20 #18 or #6 or #7 or #8 or #9 or #10 or #11  #21 #20 and #19 |
| Web of Science | ((Hip* or knee* or joint*) NEAR/3 (replace* or arthroplast* or prosth* or endoprosth*)) (Topic) and antiplatelet* or aspirin or clopidogrel or ticagrelor or prasugrel or cilostazol or dipyridamole (Topic) |

Table S1: Medical Subject Headings and free words used in the literature search across the four databases
