# Supplementary material for: The effects of continuing aspirin and clopidogrel on perioperative outcomes in primary elective total knee and hip replacement: A systematic review and meta-analysis
Source: J Orthop. 2025 Jul 24;67:369–77. doi: 10.1016/j.jor.2025.07.024 (PMC12320541; doi:10.1016/j.jor.2025.07.024)
Supplement: Multimedia component 3 [file mmc3.docx]

| First author (Year) | Selection | Comparability | Outcome | Total Score |
| --- | --- | --- | --- | --- |
| Ashkenazi (2020) | ★★★★ | ★ | ★★★ | 8 |
| Chen (2018) | ★★★★ |  | ★★★ | 7 |
| Cossetto (2012) | ★★★★ |  | ★★★ | 7 |
| Hang (2020) | ★★★★ | ★ | ★★★ | 8 |
| Li (2022) | ★★★★ | ★ | ★★★ | 8 |
| Meier (2016) | ★★★★ | ★★ | ★★★ | 9 |
| Schwab (2017) | ★★★★ | ★ | ★★★ | 8 |
| Wu (2022) | ★★★★ |  | ★★★ | 7 |

**Table S2:** Summary of judgements about each risk of bias category for each included study
